# The Hypogeous Roman Archeological Museum of Positano: Study of the Evolution of Biological Threaten and Development of Adequate Control Protocols

**DOI:** 10.3390/microorganisms12081520

**Published:** 2024-07-24

**Authors:** Federica Antonelli, Sara Iafrate, Marco Tescari, Manuel Giandomenico, Alma Kumbaric, Marco Bartolini

**Affiliations:** 1 Biology Laboratory, Central Institute for Restoration, Via di San Michele 25, 00153 Rome, Italy; alma.kumbaric@cultura.gov.it (A.K.); marco.bartolini@cultura.gov.it (M.B.); 2 Bio.Co.Ré. Lab, Via Reatina, 10, 67068 Scurcola Marsicana, Italy; 3 Mural Paintings Laboratory, Central Institute for Restoration, Via di San Michele 25, 00153 Rome, Italy; sara.iafrate@cultura.gov.it (S.I.); manuel.giandomenico@uniroma1.it (M.G.); 4 Biology Laboratory, Central Institute for Restoration, Support Staff Ales S.p.A., Via di San Michele 25, 00153 Rome, Italy; marco.tescari@cultura.gov.it; 5 Department of Science of Antiquities, Sapienza University of Rome, Piazzale Aldo Moro 5, 00185 Rome, Italy; 6 Department of Environmental Biology, Sapienza University of Rome, Piazzale Aldo Moro 5, 00185 Rome, Italy

**Keywords:** hypogeum, mural paintings, biodeterioration, cultural heritage, preservation protocols, conservation

## Abstract

Hypogea are natural or artificial spaces located underground often of great interest from an anthropological, archeological, religious, artistic, or historic point of view. Due to their features, these environments usually present conservative problems and biological colonization could be considered as one of the main threats. The present three-year study was carried out by specialists of the Central Institute for Restoration of Rome (ICR) in the hypogeous site preserved in the Roman Archeological Museum of Positano (Positano MAR) and focused on characterizing biological alterations present on the mural paintings; setting up efficient strategies and protocols for biodeterioration control; and monitoring the efficacy of direct and indirect interventions. Patinas with different morphologies were analyzed through microscopic observations, cultural analyses and next-generation sequencing. The results proved that the alterations comprised a great variety of microorganisms forming very distinct communities, differently distributed over space and time. The main taxa represented were bacteria of phyla *Pseudomonadota* and *Actinomycetota*, fungi belonging to the genus *Fusarium* and *Gliocladium*, and algae of the genus *Chlorococcum.* Preservation protocols were set up considering the alterations’ composition and included the application of biocides, limiting daily temperature changes, decreasing illuminance values on painted surfaces, and the screening of natural light sources.

## 1. Introduction

Hypogea are artificial or natural spaces located underground, usually hardly accessible, as they generally communicate with the outside environment through narrow openings. They could differ in dimensions, spatial complexity (one or more rooms), and depth in relation to the overlying bedrock or soil. Some hypogea environments were not meant to be so, their underground locations being determined by historical events, usually catastrophic (earthquakes, eruptions, and avalanches) or anthropic.

Their importance can be considered under different points of view: geological, anthropological, archeological, religious, artistic, and historic (i.e., the presence of architectural decorations, such as wall paintings, stuccoes, or carved stone reliefs). These features can be present at the same time or only one can be prevailing.

The main conservative problems affecting hypogea are due to their characteristic conformation of hardly accessible underground sites [1]. Hypogea are often dark places, characterized by scarce natural lighting, with a high humidity level, strong thermal inertia, often low or absent air circulation, and the presence of soil or other organic substances. These environmental conditions, together with the presence of artificial lighting, or sudden alterations of microclimatic parameters (linked to problems in control systems or to the presence of visitors) [2,3,4,5], create an environment favorable for the growth of microorganisms, both phototrophic and heterotrophic [5,6]. Several studies in the literature have considered the characterization of the biodeterioration phenomena of different hypogeal environments, and in recent years, thanks to the diffuse use of biomolecular tools, more and more information is gained on microbial biodiversity and on microorganism metabolic and ecological profiles [7,8,9,10,11,12]. Generally, the identified biodeteriogens are bacterial species (mainly belonging to *Actinomycetota* but also *Bacillaceae*, *Bacillota*, and *Pseudomonadota*), fungi (mainly *Sordariomycetes* and *Eurotiomycetes*), green algae (*Trebouxiophyceae*), and cyanobacteria of the class *Cyanophyceae* [5,13].

Even if studies proved that microorganisms present in such complex ecosystems are often dormant or have a slow metabolic activity [14,15], the possible biodeteriogen effect (e.g., biomineralization phenomena, the production of chelating or acid compounds, and hyphal mechanical activity) should not been undervalued [4,12,16,17,18]. For this reason, the use of indirect or direct control methods must be taken into account, and the cooperation of different expertise, particularly biologists and conservators/restores, is necessary to draw up efficient strategies for conservation and biodeterioration control and management, adopting procedures and methods that are compatible with the original materials and the conservative needs of decorated surfaces.

The present work focused on the study of the biological colonization phenomena of the archeological site preserved in the Roman Archeological Museum of Positano (Positano MAR, Salerno, Italy) and on the development of control strategies. In particular, the biological patinas present on the mural paintings were characterized by defining their macroscopic aspect and microbial composition, and their extension and location was mapped over a period of three years. Furthermore, the study attempted to find a correlation between the presence of microorganisms and the environmental parameters (relative humidity, temperature, and lighting values). Finally, part of the work was dedicated to the development of efficient conservation procedures and strategies compatible with painting materials and of a maintenance plan for the correct conservation of paintings that could match the needs linked to the fruition of the site by visitors.

## 2. Materials and Methods

### 2.1. The Roman Archeological Museum of Positano

The Roman Archeological Museum of Positano (Positano MAR) hosts the hypogeous roman *villa* and preserves a valuable testimony of Roman wall paintings [19,20,21]. Dating back to the 1st century AD, these frescoes belong to the fourth Pompeian style and adorned the walls of the room that was originally the *triclinium* of the *villa maritima* in Positano’s bay area. Like other *villas* in the Vesuvian region, the one in Positano suffered damage from the earthquake of 62 AD and the volcanic eruption of Mount Vesuvius in 79 AD. Following the eruption, rains caused the detachment of volcanic deposits landed on the hillslope surrounding Positano city, leading to a landslide that buried the *villa*. The roman *villa* remained hidden until 1758 when its presence was mentioned by Karl Weber during the description of the excavations conducted near the bell tower of Positano’s main church. Systematic excavations started in 2003 and led to the rediscovery of the villa’s *triclinium* and its mural paintings, unearthing significant portions of the *triclinium* walls.

Structural consolidation works and restoration interventions led to the opening of the Positano MAR in 2018, when the hypogeous *villa* became open to the public.

The museum is divided into two levels, corresponding to historical stratifications: the lower level (11 m below street level) features the Roman *triclinium*, while the upper level corresponds to the upper crypt built in the 18th century on top of the Roman *villa*, which at that time was no longer visible after being buried under volcanic mud. The visitor’s path winds through a walkway that ends in a staircase with glass steps, allowing visitors to approach the Roman paintings on the lower level.

To assure the proper conservation of the wall paintings and avoid the conservative drawbacks due to the visitors’ presence, the environmental parameters of the underground rooms are maintained constant thanks to a centralized climate control and conditioning system equipped with a detection station that guarantees the remote control of the hypogeum parameters. Air conditioning is also aimed at reducing the concentration of environmental radon and carbon dioxide released by visitors. The number of visitors (10) and their visiting time (30 min every two hours) have been set to minimize their impact on microclimatic stability.

Additionally, a timed lighting system was installed with dimmable light intensity and multiple light sources to guarantee a homogeneous distribution of the illuminance value of the painted surface. The artificial lighting sources are six different spotlights distributed on two different levels, three of them for the upper part of the paintings (distance from the painted surface of about 3.5 m) and three for the lower part of the paintings (distance from the painted surfaces between 0.5 and 1.5 m).

At the beginning of the study (2021), when specialists from the Central Institute for Restoration (ICR) made their first inspection on request of the Sovrintendenza Archeologia belle arti e paesaggio per le Provincie di Salerno e Avellino and the Positano Municipality, the climatic parameters characterizing the hypogeous context were the following: temperature values between 16.5 °C and 19.5 °C and very high and constant relative humidity levels (about 90%). Light intensity values showed an average value of 120 lux.

### 2.2. Biological Patinas

**Figure 1:**
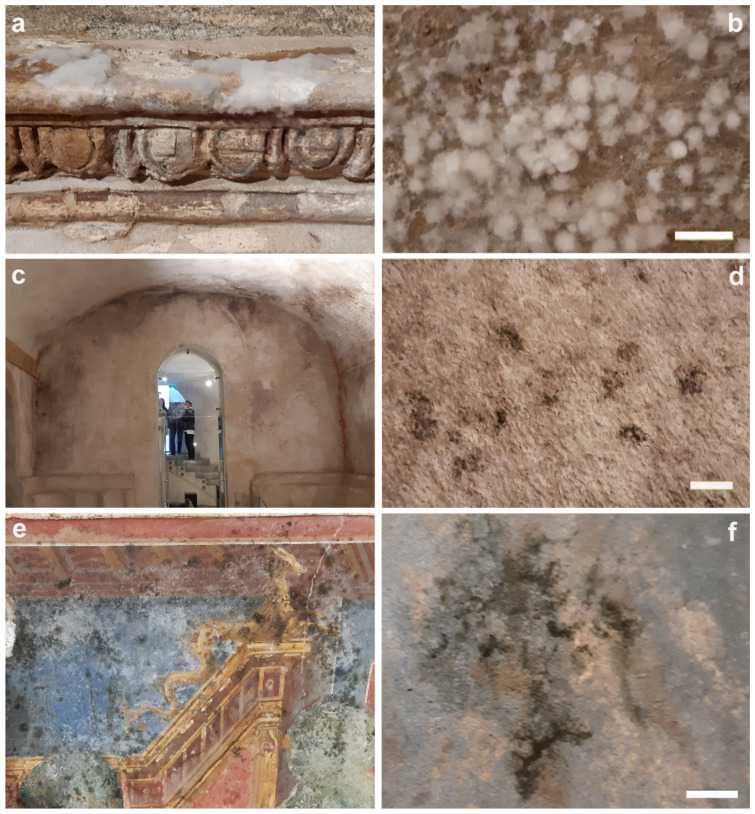
Aspects of the analyzed patinas. (**a**,**b**) FW—filamentous white patina; (**c**,**d**) BS—black spots; (**e**,**f**) BF—black filamentous patina. Scale bar: 0.5 cm.

**Figure 2:**
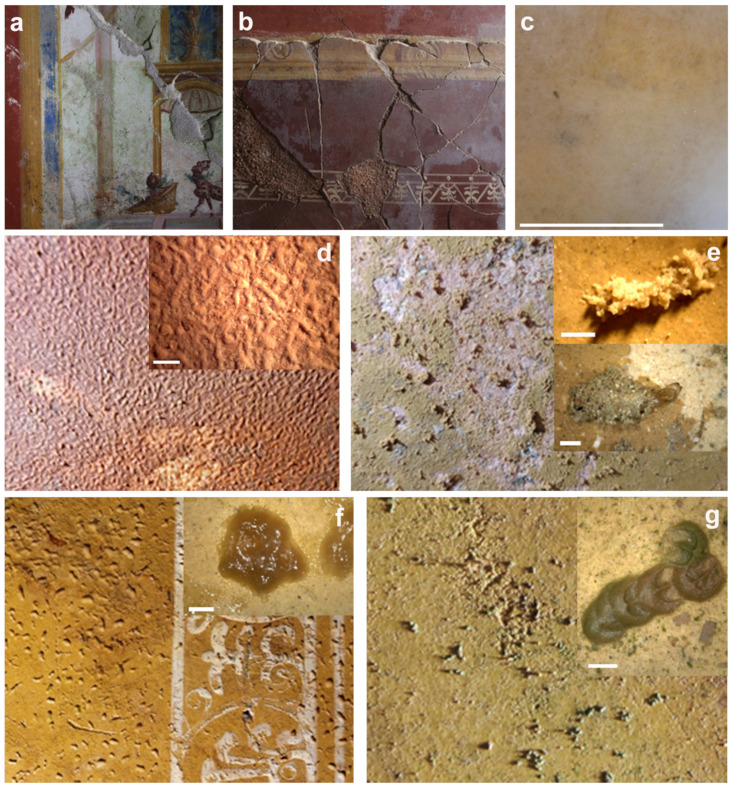
Aspects of the analyzed patinas. (**a**) GP—green patina; (**b**,**c**) BsF—brown semitransparent film; (**d**) FT—soft thin filamentous white patina; (**e**) GC—granular clumps; (**f**) JC—jelly clumps; (**g**) CC—compact clumps. Scale bar: 1 mm.

**Figure 3:**
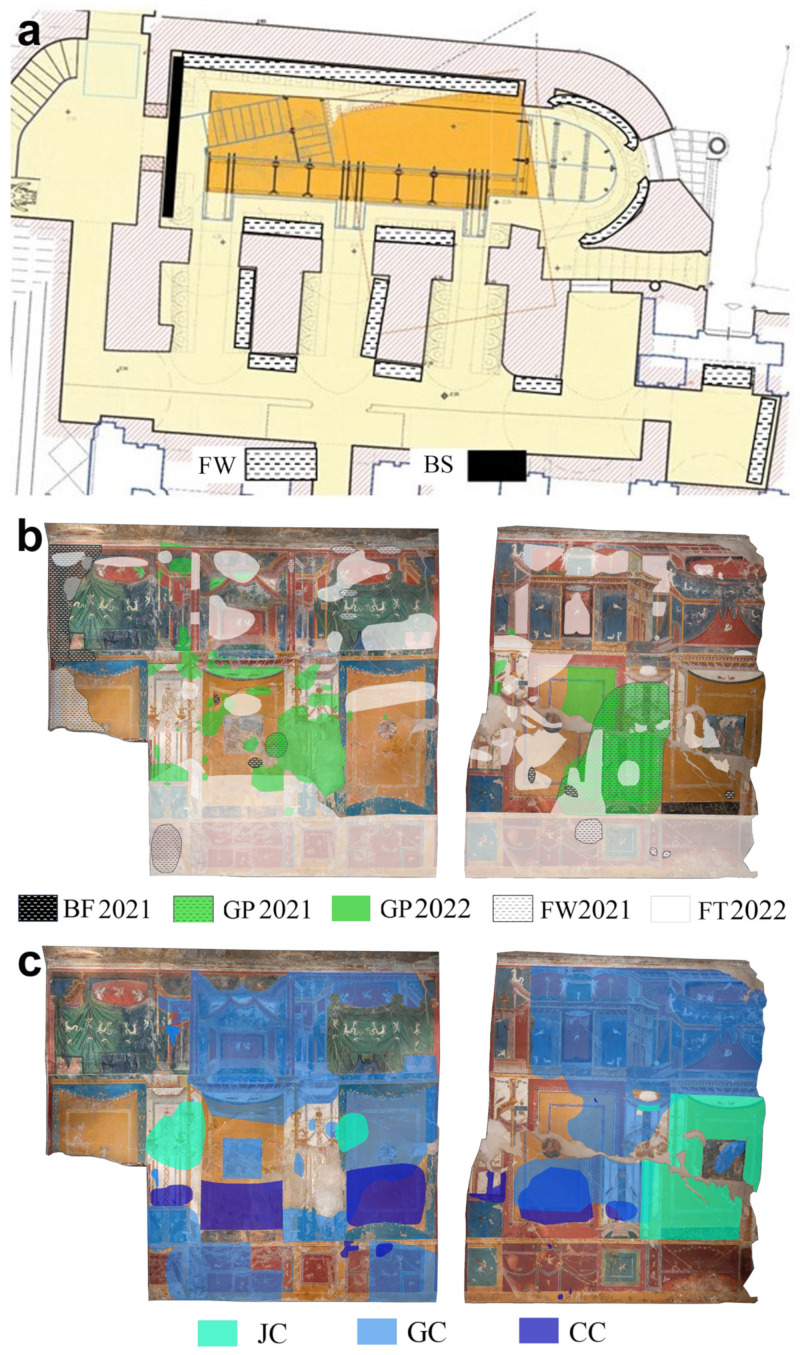
Maps reporting the distribution of the analyzed patinas on the surfaces of the upper crypt (**a**) and of the Roman villa (**b**,**c**).

The description of the nine patinas analyzed throughout the study is reported in Table 1. Their aspects and distribution on the surfaces of the Roman *villa* and of the upper crypt is reported in Figure 1, Figure 2 and Figure 3.

### 2.3. The Study

**Figure 4:**
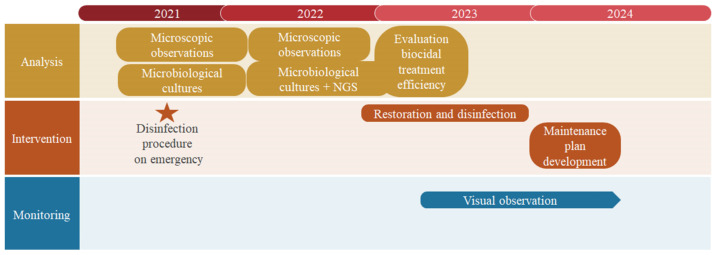
Timetable summarizing the activities carried out during the study.

The study carried out in the Positano MAR began in 2021 and lasted until 2024. The activities performed during these three years are summarized in Figure 4. As it can be seen, the characterization of the biological patinas was strictly interconnected with the development and application of conservation procedures. To face the biological colonization of painting surfaces, both direct and indirect methods have been drawn up (see Section 4).

The patinas described in Section 2.2 were analyzed during two analytical campaigns.

The first campaign, carried out in 2021, was necessary because of the presence of substantial colonization on the crypt’s surface and on the upper part of the *villa’s* mural paintings (patinas FW, BS, and BF) (Figure 1 and Figure 3). The development of the patinas was related to a malfunctioning of the air conditioning system that led to an uncontrolled alteration of thermohygrometric conditions. In this emergency phase, the characterization studies were mainly aimed at confirming the presence of microorganisms and to their identification in order to draw up an efficient procedure for the painted surfaces’ disinfection to prevent the diffusion of the biodeteriogen colonization and to limit the damages produced by microorganisms.

The second analytical campaign began in March 2022 when patinas different from those already analyzed were detected during the study of the conservation status of the painted surfaces of the *villa* aimed at the development of conservation procedures.

Apart from the green patina (GP) (Figure 2a), easily referable to the presence of photosynthetic microflora, other alterations (BsF, GC, JC, and CC) (Figure 2b–g) were quite peculiar and uncommon and not easily ascribable to biological colonization. Their morphology was similar to that of crystallized soluble salts (e.g., subflorescence), although disgregation of the painted layer and the carbonatic support, damages frequently combined with salt decay, were not detectable. Furthermore, conductivity measurements carried out on the surface revealed very low values not compatible with the presence of soluble salt. This evidence led to the hypothesis of a biological origin of the alterations that underwent further investigation through different analytical techniques.

The analytical methods were different depending on the type of alteration (Table 2). The alterations most easily attributable to a biological origin were analyzed by microscopic observation (MO) and microbiological cultures (MC). The community composition of atypical alterations was also investigated through next-generation sequencing (NGS). During 2023, the restorers carried out the restoration of the wall paintings and monitored the state of the surfaces over one year (March 2023–April 2024) through visual observation and portable video microscopes (Dino-lite AD 7013MZT, AnMo Electronics Corporation, Taipei, Taiwan) in order to highlight any reappearance of the alterations.

### 2.4. Microscopic and Microbiological Cultures

Patinas were investigated in situ with portable digital microscopes (Dino-lite AD 7013MZT). The sampling mode was different depending on the laboratory analysis to be carried out. For microscopic observations, patinas were sampled by scraping the surface with a sterile scalpel or using fungi tape. Here and for NGS analyses (see Section 2.5, scalpel sampling was carried out by restorers taking only biomass without damaging the painted surface. Sampled materials were observed as unstained wet mounts through an optical microscope (Zeiss Axio Imager M2, Oberkochen, Germany) equipped with digital camera.

For microbiological cultures, patinas were sampled with a sterile swab. Analyses were carried out on three cultural media suited for different microorganisms: Mycological Agar (BD DIFCO, Mississauga, ON, Canada) Plate Count Agar (PCA) (Oxoid, Hampshire, UK), and Actinomycetes Isolation Agar (BD DIFCO). After transferring the sampled patinas onto the culture media, the plates were then incubated at 27 ± 2 °C for 14 days. Microorganisms were identified through the observation of macroscopic features of the colonies and microscopic taxonomical characteristics.

### 2.5. Sampling, DNA Extraction, Amplification, Sequencing, and Data Analysis

NGS analyses were performed during the second analytical campaign in March 2022. The sampling of alterations was conducted by collecting surface material using a sterile scalpel. Samples were stored in a cold storage unit on the same day of sampling until DNA extraction. The extraction of DNA was performed with a DNeasy^®^ Powersoil^®^ Pro Kit (QIAGEN, Hilden, Germany), following the manufacturer’s instructions. The quality of the extracted DNA was determined by measuring the A260/A280 ratio in NanoSNAP™ microvolume spectrophotometer (Thermo Fisher Scientific, Waltham, MA, USA).

Partial 16S rRNA gene sequences were amplified from extracted DNA using the primer pair Probio_Uni and/Probio_Rev, targeting the V3 region of the 16S rRNA gene sequence [22]. 16S rRNA gene amplification and amplicon checks were carried out as previously described [22]. 16S rRNA gene sequencing was performed using a MiSeq (Illumina, San Diego, CA, USA) at the DNA sequencing facility of GenProbio s.r.l. (Parma, Italy) (www.genprobio.com, accessed on 18 July 2024) according to the protocol previously reported [22]. Following sequencing, the .fastq files were processed using a custom script based on the QIIME2 software suite (https://qiime2.org/, accessed on 18 July 2024) [23,24]. Paired-end read pairs were assembled to reconstruct the complete Probio_Uni/Probio_Rev amplicons. Quality control retained sequences with a length between 140 and 400 bp and mean sequence quality score > 20, while sequences with homopolymers > 7 bp and mismatched primers were omitted. To calculate downstream diversity measures (alpha and beta diversity indices, Unifrac analysis), 16S rRNA Amplicon Sequence Variants (ASVs) were defined at 100% sequence homology using DADA2 (https://benjjneb.github.io/dada2/index.html, accessed on 18 July 2024) [25]; ASVs not encompassing at least two sequences of the same sample were removed. Notably, this approach allows highly distinctive taxonomic classification at single nucleotide accuracy [25]. All reads were classified to the lowest possible taxonomic rank using QIIME2 [23,24] and a reference dataset from the SILVA database (https://www.arb-silva.de/, accessed on 18 July 2024) [26]. Biodiversity within a given sample (alpha-diversity) was calculated with Chao1 and Shannon indexes calculated for ten sub-samplings of sequenced read pools and represented by rarefaction curves (Figure S1). Similarities between samples (beta-diversity) were calculated by weighted uniFrac [27]. The range of similarities is calculated between values 0 and 1. Principal Coordinates Analysis (PCoA) representations of beta-diversity were performed using QIIME2 [23,24].

The profiling of known fungal species was performed employing the primer pair BITS/B58S3 [28] targeting fungal ITS. Sequencing was performed using a MiSeq (Illumina) at the DNA sequencing facility of GenProbio s.r.l. (www.genprobio.com, accessed on 18 July 2024) according to the protocol previously reported [22]. As previous described for 16S rRNA gene sequence data analysis, the .fastq files were processed using a custom script based on the QIIME2 software suite. In detail, quality control retained sequences with a mean sequence quality score > 15, while sequences with mismatched primers were omitted. In order to calculate fungal taxonomy, ITS rRNA ASVs at 100% sequence homology using DADA2 and ASVs not encompassing at least two sequences of the same sample were removed. All reads were classified to the lowest possible taxonomic rank using QIIME2 and a reference dataset from the UNITE database (https://unite.ut.ee/, accessed on 18 July 2024) [29]. Due to the shortness of Illumina-produced fungal ITS sequences, identification of fungi was limited to the genus as it is the lowest taxonomic level for which the system is reliable.

Similarities between samples (beta-diversity) were calculated using Bray–Curtis dissimilarity. The range of similarities is calculated between values 0 and 1. PCoA representations of beta-diversity were performed using QIIME2.

The raw reads were deposited into the NCBI Sequence Read Archive (SRA) database (https://www.ncbi.nlm.nih.gov/sra, accessed on 18 July 2024) (SRA accession no.: PRJNA1129806).

### 2.6. Evaluation of Biocidal Treatment Efficacy

In 2022, the patinas present on the painted surfaces of the *villa* (GP, GC, JC, CC, and BsF) were treated with a biocide based on Quaternary Ammonium Compounds (QACs), Preventol RI50 (Lanxess, Cologne, Germany), diluted in deionized water at 5% in concentration, and applied once by brushing interposing sheets of Japanese paper to avoid the contact of the brush with the fragile paint layer. To verify whether this procedure achieved an adequate level of effectiveness, the viable microbial load was analyzed by evaluating ATP through bioluminescence tests one week after treatment. Cells viability was estimated with a Hygiena ATP Hygiene Monitoring System-SystemSure Plus Luminometer and UltraSnap swabs (Hygiena LLC, Camarillo, CA, USA). Swabs were rubbed on a 1 cm^2^ area and the analyses were performed immediately after sampling. The results are expressed as the percent decrease of Relative Light Units (RLU) between treated (TR) and untreated (UN) patina.

## 3. Results

### 3.1. Microscopic and Microbiological Analyses

**Figure 5:**
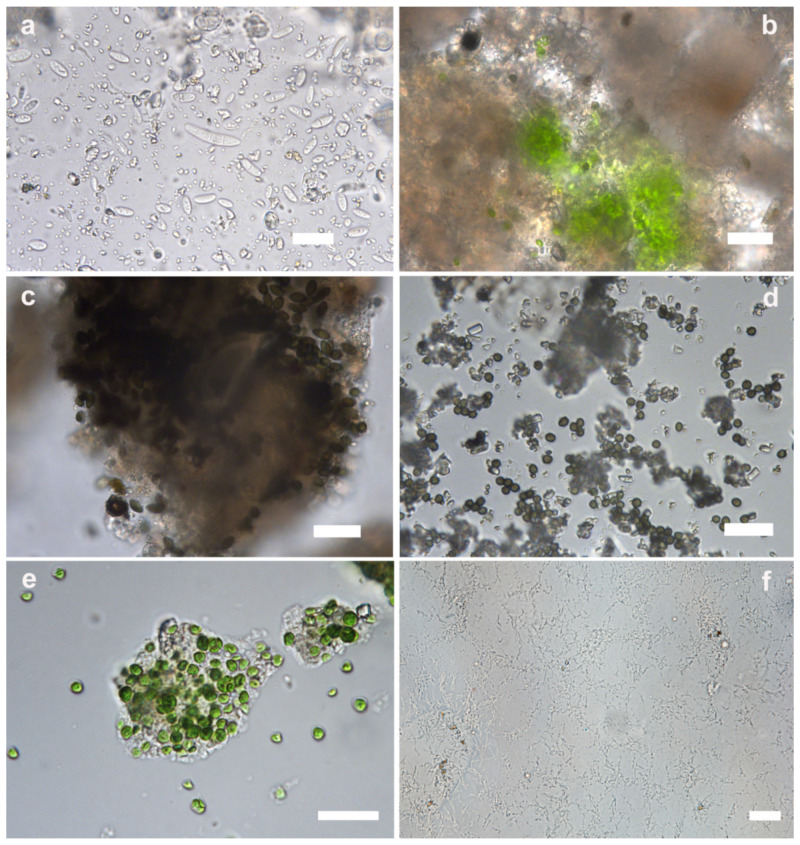
Microscopic images of the analyzed patinas. (**a**) FW, sickle-shaped septate macroconidia and single-celled microconidia belonging to a genus-like *Fusarium*; (**b**,**c**) BS, clusters of algal cells and fungal conidia with dark pigmentation; (**d**) BF, dark pigmented fungal conidia; (**e**) GP, green algae belonging to the genus *Chlorococcum*; (**f**) FT, thin filamentous structures referable to *Actinomycetota*. Scale bar: 20 µm.

Optical microscope observations highlighted that the filamentous white patina (FW) was composed of hyphae and conidia of an hyphomycete attributable to a species of the genus-like *Fusarium*. Two of the three types of asexual spores produced by fusarioid species of fungi, micro and macroconidia, were clearly distinguishable (Figure 5a). Microconidia were single-celled, ≤10 µm in length, and globose, oval, or reniform. Macroconidia were fusiform or sickle-shaped, multi-celled by transverse septa, and more than 20 µm in length. Microbiological analyses confirmed the presence of this sole type of fungi.

Wet-mount observations of black spots (BS) showed the presence of photosynthesizing microflora referable to the taxon *Chlorophyta* and of some fungal conidia with dark pigmentation (Figure 5b,c). Unfortunately, it was not possible to identify these microorganisms due to the lack of taxonomical elements.

In the black filamentous patina (BF) sample, numerous dark pigmented fungal conidia and fragments of colorless hyphae were detected (Figure 5d), but the lack of morphological elements did not allow a more precise identification for taxonomic recognition. Rare colorless sickle-shaped conidia, morphologically referable to *Fusarium* genus, were also observed. Cultural analyses resulted in the development of only *Fusarium*-like fungi. The result could be related to the high growth rate of these species [30], which may have inhibited the development of other fungi.

The green patina (GP) was mainly composed of a green alga (*Chlorophyta*) belonging to the genus *Chlorococcum* (Figure 5e). The cells were ellipsoidal to spherical and vary in size. They were mostly solitary, and groups of few aggregated cells were rarely observed. Rare cells of a cyanobacterium identified as *Chroococcus* sp. were also observed.

Optical microscope analysis of the soft, thin filamentous white patina (FT) highlighted the presence of thin filamentous structures morphologically referable to *Actinomycetota* (Figure 5f). The presence of these microorganisms was confirmed by microbiological investigations, and their identification was obtained through NGS (see Section 3.2).

**Figure 6:**
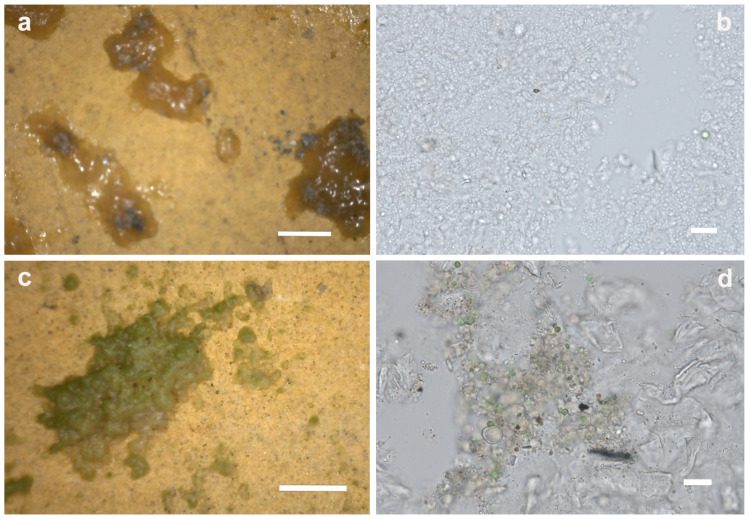
Portable digital microscope (**a**,**c**) and optical microscope (**b**,**d**) images of the analyzed patinas. (**a**) JC, presence of pigment grains in the patina; (**b**) JC, small cells referable to non-pigmented mucilaginous heterotrophic bacteria; (**c**) GC, granular clump covered by a green patina; (**d**) GC, colorless amorphous substance, probably attributable to EPS and the acrylic resin residues. Scale bar: (**a**,**c**): 1 mm, (**b**,**d**): 20 µm.

The observation of jelly clumps (JC) through portable digital microscopy showed the presence of pigment grains (Figure 6a). Optical microscope analyses of the biofilm highlighted the presence of numerous small cells (<1 µm) referable to non-pigmented mucilaginous heterotrophic bacteria (Figure 6b). The cells were held together by a colorless matrix attributable to extracellular polymeric substances (EPS) produced by the cells themselves during the development of the microbial biofilm. Some cells, referable to *Chlorophyceae* and *Bacillariophyceae*, and a colorless amorphous substance were also detected. The latter was probably attributable to residues of acrylic resin-based restoration products used in previous interventions, as proven by FT-IR analyses.

On field microscope observation, granular clumps (GC) appeared to consist of granular particles held together by a gelatinous substance not always clearly visible. These clumps exhibited a beige or reddish-brown coloration, unrelated to the underlying surface color. In some cases, residues of biological material were found within the clusters. These clusters sometimes were covered by a green patina (Figure 6c). Optical microscopic observation of GC and CC samples revealed strong similarities in their structure and composition in spite of morphological differences initially seen by the naked eye.

In all the samples, a colorless amorphous substance, probably attributable to extracellular polymeric substances (EPS) and the acrylic resin residues already found in JC, was detected (Figure 6d). Furthermore, brownish particles with irregular shapes (not referable to biological structures) and green cells referable to coccal microalgae (division *Chlorophyta*) were observed. The quantity of algal cells varied depending on the position of the sampling; indeed, they were very abundant in areas characterized by high light intensity.

### 3.2. Microbial Diversity and Community Structure

Microbial diversity in samples was investigated both for bacteria and fungi, respectively targeting the 16S rRNA gene V3 region and fungal ITS.

Bacterial profiling was grounded on a total of 262,870 paired-ends reads, with an average of 43,812 reads per sample. Processing data through DADA2 resulted in a total of 832 bacterial ASVs (representing 99.47% of reads). Rarefaction curves (Figure S1) were calculated and Chao1 and Shannon indexes were determined. The saturation plateau was reached for both indexes and in all samples, indicating adequate coverage of the biodiversity present within the samples. Among the six samples, CC showed the highest ASV richness (Chao1 = 339) and FT the lowest (Chao1 = 119). Based on the Shannon index, CC showed the highest ASV diversity (Shannon = 5.911) while JC2 the lowest (Shannon = 4.404). Furthermore, 554 unique ASVs (66.59% of total ASVs) were identified from the six samples, representing 25.86% of the total sequences. Conversely, 278 ASVs were present in more than one sample, accounting for 75.14% of the total sequences, meanwhile only three ASVs were always present in samples, accounting for 5.32% of the total. These findings suggest that few taxa are prevalent in more than one sample, meanwhile PCoA representations of beta-diversity shows similarities between samples (Figure S2). Indeed, samples JC1 and JC2 are clustered together, as are the couple GC and CC, while samples BsF and FT are distant points in the graph.

Fungal profiling was grounded on a total of 12,037 paired-ends reads with an average of 2006 reads per sample. Data analysis through DADA2 resulted in a total of 31 fungal ASVs, accounting for 100% of the reads. There were 17 unique fungal ASVs, accounting for 3.67% of the total sequences, while the ASVs present in more than one sample represented 96.33% of the total. Any ASVs are shared in all six samples. Additionally for fungi, the findings suggest that few taxa are prevalent in more than one sample. The PCoA (Figure S2) shows a cluster of four samples (JC2, GC, CC, and BsF) that are similar, while FT and JC1 are distant from the cluster and from each other.

**Figure 7:**
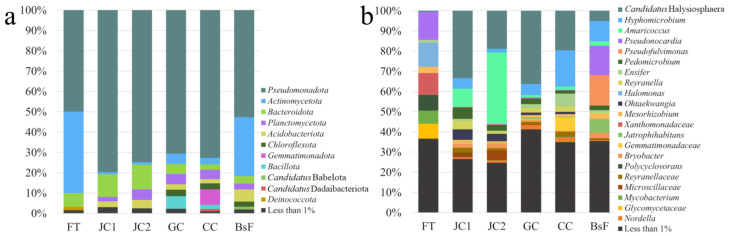
Bacterial community composition obtained by 16S rRNA gene sequencing (V3 region) of six samples: FT, JC1, JC2, GC, CC, and BsF. The relative abundances are given at the phylum level (**a**) and genus/family level (**b**). All ASVs with an abundance lower than 1% are grouped together and displayed as “Less than 1%”.

The bacterial community structure was composed of 23 phyla, 183 families, and 265 genera in the six samples. The predominant bacterial phyla, in total relative abundances order, were as follows: *Pseudomonadota* (66.30%), *Actinomycetota* (13.87%), *Bacteroidota* (6.63%), *Planctomycetota* (3.27%), *Acidobacteriota* (3.04%), *Chloroflexota* (1.71%), *Gemmatimonadota* (1.68%), and *Bacillota* (1.51%), followed by minor phyla (<1%) representing 1.98% of the total sequences. *Pseudomonadota*, *Actinomycetota*, and *Bacteroidota* were the only phyla present in all samples, with *Pseudomonadota* as the most prevalent phylum in all samples, ranging from 79.70% in JC1 to 49.96% in FT (Figure 7a). Eight bacterial genera were shared by all samples with a relative abundance ≥ 1%: *Candidatus* Halysiosphaera (18.24%), *Hyphomicrobium* (7.42%), *Pseudonocardia* (5.35%), *Ensifer* (2.14%), *Mesorhizobium* (1.76%), *Jatrophihabitans* (1.66%), *Bryobacter* (1.41%), and *Mycobacterium* (1.22%). Even though there were various genera shared among the samples, the relative abundance in the different samples was highly variable. For example, *Candidatus* Halysiosphaera was the prevalent genus but it ranged from 36.31% in GC to 0.51% in FT (Figure 7b).

**Figure 8:**
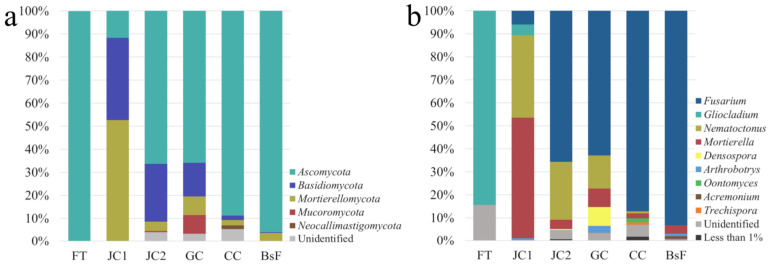
Fungal community composition obtained by ITS sequencing of six samples: FT, JC1, JC2, GC, CC, and BsF. The relative abundances are given at the phylum level (**a**) and genus level (**b**). All ASVs with an abundance lower than 1% are grouped together and displayed as “Less than 1%”.

On the other hand, the fungal community structure was composed of five phyla, twelve families, and fourteen genera in the six samples. Ascomycota was the predominant phylum, accounting for 82.30% of the total sequences, and the only phylum present in all six samples. Other phyla, accounting for more than 1% and were not present in all samples, were as follows: *Basidiomycota* (8.05%), *Mortierellomycota* (6.80%), and *Mucoromycota* (1.38%) (Figure 8a). Any fungal genus was shared by all samples. *Fusarium* was the most abundant genus, accounting for 79.35% of the total sequences. Other genera with a relative abundance ≥ 1% were *Nematoctonus* (7.77%), *Mortierella* (6.80%), *Densospora* (1.38%), and *Arthrobotrys* (1.25%) (Figure 8b). Notably, the genus *Gliocladium*, which accounted for 84.44% of the relative abundance in the FT sample, represented only the 0.56% of the total sequences. This discrepancy is due to the small number of sequences obtained for the FT sample.

### 3.3. Evaluation of Biocidal Treatment Efficacy

Results of ATP bioluminescence assays showed that biocidal treatment was effective on almost all the patinas. In particular, a marked decrease in cell viability was obtained exceeding 96% for JC and 99% for GC, CC, and GP (Table 3).

The treatment on BsF alteration was less efficient because the previous removal of the biomass could not be performed as the disgregation and powdering of the paint layer did not allow the delicate mechanical stress necessary for the cleaning.

## 4. Discussion

The analyses carried out during the three-year study highlighted that the biological patinas present on the surfaces of the artifacts preserved in the Roman Archeological Museum of Positano (Positano MAR) were very variable both in terms of composition and spatial distribution.

The results of microscopic observations and of next-generation sequencing (NGS) showed that alterations with peculiar morphologies not clearly referable to biological causes, related to the development of bacteria, that were predominant. Indeed, bacteria-related sequences were 21 times the number of the sequences attributable to fungi. Bacterial community structure showed differences between samples from the phylum level. Four different groups have been identified. The presence and abundance of phyla in samples showed similarities between JC1 and JC2 and between GC and CC, while FT and BsF showed differences between each other and with other samples. *Pseudomonadota* was clearly the prevalent phylum in all samples, while *Actinomycetota* was the second most abundant phylum and the most variable in abundance. Both phyla are usually found in hypogeum [13].

Bacterial community structures at the lower taxonomic level (family and genus) showed clearer differences, maintaining the same division in the group seen above. The FT sample was the most different in composition among the samples, with *Pseudonocardia*, *Halomonas*, and *Xanthomonadaceae* as the prevalent taxa. *Pseudonocardia* was present in all samples, but in FT and BsF, its relative abundance was >10%, while in other samples it was <1%. Conversely, *Halomonas* and members of *Xanthomonadaceae* family were present only in FT samples. Concerning the genera *Pseudonocardia* and *Halomonas*, they have been detected in other hypogeum contexts: in the white colonization in quarries with paleolithic wall paintings [31] and in the mural wall painting in the Crypt of the Original Sin (Matera, Italy) [32].

In other samples, the most abundant genus was *Candidatus* Halysiosphaera. This non-culturable genus is known as filamentous bacteria, common in wastewater treatment plants of different kinds of industries, often causing bulking problems [33,34]. Among the few available pieces of information about *Ca*. Halysiosphaera, it is known that it is a floc-forming bacteria in water [35]. Floc-forming abilities could trigger the formation of clumps on the frescoes, and this is consistent with the morphology of samples (JC1, JC2, GC, and CC) that appear as clumps in which *Ca*. Halysiosphaera is prevalent. Even if in our samples it was highly represented, *Ca.* Halysiosphaera is for the first time found associated with a wall painting or in a hypogeum.

Second in relative abundance was the *Hyphomicrobium* genus, which was present in all samples ranging from 0.13% in FT to 17.93% in GC. Members of the *Hyphomicrobium* genus were found in cave churches [36], in hypogea [13,37], and in wastewater ponds [38]. Third in relative abundance and present in all samples, excluding FT, was *Amaricoccus*, which ranged between 1.14% in GC to 35.35% in JC2. As for *Ca*. Halysiosphaera, *Amaricoccus* were found in sludge and wastewater treatment plants [39], but it seems to be ubiquitous: from blood [40] to Antarctic soil samples [41], and passing by cleanrooms [42]. *Amaricoccus* is known as the aerobic chemoheterotroph genus [39].

**Figure 9:**
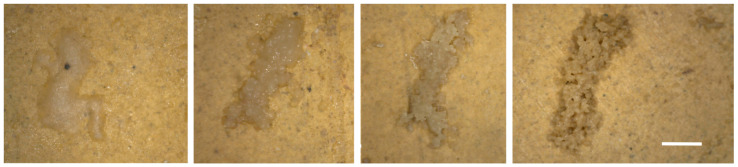
Increasing grade of hydration of clumps from JG to GC according to their proximity to the protruding bank rock. Scale bar: 1 mm.

Considering NGS results, the differences in JC, GC, and CC bacterial composition cannot satisfactorily explain their different morphologies, which instead are more likely related to different grades of hydration of the clumps. JC, which can be considered the most hydrated form, was found only in areas of the painted surface protected from the microclimatic system airflow by the protruding volcanic bank rock (Figure 9). Corroborating this hypothesis, the presence in JC alterations of *Bacillariophyta*, typically growing in constantly wet habitat [43], must be noticed. These algae were not present in GC and CC alterations.

The analyses revealed that fungi were also well represented in the analyzed patinas, and their presence was confirmed through all the applied techniques in both the analytical campaigns. However, it must be highlighted that, in the light of results of NGS analysis, the prevalence of fungi seems to be decreased over time. In fact, the sequencing in all samples revealed a low quantity of sequences referable to the kingdom *Fungi*. These results could be attributed to the effect of conservative activities carried out in the hypogeum (see below for further details). In fact, patinas that had developed in 2021, after the malfunctioning of the air conditioning system, were predominantly attributable to fungal species, which were present in all the analyzed samples. Species belonging to the *Fusarium* genus seemed to be the only component of the FW patina, and spores attributable to these fungi were also detected in BF. The *Fusarium* genus was detected in the second analytical campaign through NGS in five of the six analyzed samples (JC1, JC2, GC, CC, and BsF). This genus is widely known in the field of cave and hypogea conservation. In particular, *F. solani* has been one of the main candidates responsible for fungal outbreaks in the cave of Lascaux and in the Castañar Cave [10,11,44,45,46], and other species belonging to this genus were isolated in hypogeus catacombs both as airborne and biofilm-associated microorganisms [47,48,49] and on mural paintings in various confined and non-confined hypogean environments [50,51].

Among the other more represented fungal genera identified in the NGS analysis, *Gliocladium* spp. are ascomycetes belonging to the family *Trichospaeriaceae* (order *Trichospaeriales*). Species belonging to this genus are known as soil-borne fungi, also found in cave environments [52] or are isolated from fern and flowering plants [53,54]. Some *Gliocladium* spp. are reported as endophytic fungi suitable for use in cropping to regulate plant heat stress [55]. The genera *Nematoctonus*, *Arthrobotrys*, and *Densospora* comprise nematode trapping fungi [56,57,58,59]. The presence of these predators is not surprising, since nematode specimens have been observed in wet mounts of some of the studied patinas.

*Mortierella* is a genus of mucormycetes belonging to the family *Mortierellaceae* (order *Mortierellales*). Species belonging to this family are commonly isolated from soil or organic debris, usually of vegetal nature, in every continent, including Antarctica [60,61]. In particular, species of the genus *Mortierella* are important members of the microbial soil community [62] that have also been isolated from cave sediments [52] and in bat guano in caves [63,64,65]. Some *Mortierella* spp. have commercial interest for being oleaginous fungi that are able to produce lipids in the form of triacylglycerols [61], in some cases accounting for up to 50% of their dry weight [66]; the fatty acids produced protect the mycelium against low temperatures, making those species psychrotolerant [67,68] and ice-nucleation-active (INA) [69]. In the field of the preservation of Cultural Heritage, fungi of the genus *Mortierella* have been isolated from a tissue artifact recovered from sealer sites in the South Shetland Islands (Antarctica) and stored between 8 °C and 10 °C [70] and from mural paintings in various confined and non-confined hypogean environments [50,51]. The species *M. alpina* has also been isolated and identified in the Castañar Cave. Together with *Fusarium solani* and *Fusarium oxysporium*, it has been considered a persistent species, being present during all the three studied fungal outbreaks in the cave, despite the preservation strategies adopted [10,11,45].

In addition to bacteria and fungi, photosynthetic microorganisms were identified in patinas BS, GP, and JC, mainly belonging to the classes *Chlorophyceae* and *Bacillariophyceae*. Phototrophs are often present in hypogeal environments in the presence of a light source, since the high environmental humidity favors these microorganisms. In particular, the presence of *Bacillariophyceae* is indicative of a constantly wet surface condition. Green algae of the genus *Chlorococcum* are frequently detected on stone materials in hypogeal environments [71].

As stated, the present work was focused not only on the characterization of the biological patinas but also on developing efficient conservation procedures and strategies compatible with painting materials and a maintenance plan for the correct conservation of paintings. As the results of the investigations showed that the alterations found on the surfaces were related to the development of microorganisms, a biodeteriogen control strategy was drawn up, including both direct methods, aimed at microorganism devitalization and disinfection treatment, and indirect methods, based on the modification of the environmental parameters, in order to prevent new colonization (Table 4).

Since the study was divided in multiple phases over three years and different biological patinas had to be faced over time, the conservation procedures were set up time after time, depending on the features and needs of the different microorganisms detected by the analytical campaign and their potential risk of damage for the painted surface or the health of visitors.

The first intervention took place in 2021 and was mainly aimed at treating the fungal outbreak (patinas FW and BF) caused by an uncontrolled alteration of thermohygrometric conditions due to a malfunctioning of the air conditioning system. In light of the high biodeteriogen potential of fungi and taking into account that many species belonging to the genus *Fusarium* are associated with human mycotoxicosis and able to cause disease in humans [72], an emergency treatment was carried out, consisting in the application of a biocide on all surfaces affected by fungal growths. A biocide based on QACs was applied twice by spraying to avoid mechanical stresses on the painted surfaces. Some indirect control strategies were also developed to prevent new fungal colonization, which was based on lowering temperature values to 15 °C and limiting the daily temperature changes in the intervals recommended by the Italian Ministry of Cultural Heritage Museum Standards Recommendations [73] and EN16873:2016 [74]. To this end, the air-conditioning system was upgraded, and the visitor pathway was modified in order to keep the second access door to the crypt and triclinium rooms constantly closed to make the entrance hall of the hypogeum work as an airlock between the outside and the hypogeous environment. This was fundamental to prevent the air flux coming from the outside (35 °C in summer) entering directly in the hypogeum and modifying environmental parameters.

The monitoring phase following this emergency intervention revealed that the fungal patinas FW and BF, and BS, were no longer visible. Nonetheless, analyses carried out during the second analytical campaign showed that, even if less abundant with respect to other microorganisms, fungi were still present on the painted surfaces. This suggests that the biocidal treatment and the designed strategies were effective in controlling the colonization, drastically reducing the fungal presence in the environment, but were not able to totally eradicate these microorganisms, confirming the persistent nature of *Fusarium* already reported in other studies [10,11]. Furthermore, the low temperatures might have selected psychrotolerant species, as suggested by the presence of *Mortierella* spp.

The monitoring also highlighted that GP was still clearly visible and had colonized a broader area of the *triclinium* mural painting (Figure 3). The broadening of the photosynthetic microorganisms on the wall paintings of the *triclinium* was determined by an increased water content of the porous surface due to condensation phenomena caused by the decreasing of temperature values (from 18 °C to 15 °C). In this case, the indirect strategy consisted in decreasing the illuminance values on the painted surface from an average value of 120 lux to an average value of approximately 50 lux, which is the minimum illuminance value required to guarantee proper color perception according to recommendations of the Italian Ministry of Cultural Heritage Museum Standards Recommendations [73]. In addition, all natural light sources, such as openings and windows of the upper crypt, were screened by applying specific filters on the glass, thus decreasing the intensity of natural light entering the hypogeous rooms.

During the second analytical campaign (March 2022), new protocols were developed to treat the GP and peculiar patinas detected during the study of the conservation status of the painted surfaces of the *villa* (GC, JC, CC, and BsF), which were identified as bacterial patina by the analytical campaign. Considering the consistency of the bacterial biomass present on the surface and its thickness, the treatment procedure was drawn up by considering an initial mechanical removal of patinas followed by biocide application. This procedure was considered appropriate since the patinas were mainly composed of bacteria, so there was no risk in spreading spores or vital propagules in the environment as it would have happened with fungi. The mechanical intervention was fundamental for reducing the quantity of vital biomass on which the biocide had to act, allowing it to reach a good level of disinfection with only one biocide application, in the perspective of economic and ecological sustainability of the intervention. The control of the treatment efficacy performed by ATP analysis confirmed that the drawn-up procedure was appropriate and allowed the obtaining of satisfactory results in term of cell devitalization.

After the end of the restoration intervention on mural paintings, the surfaces were monitored for one year. No significant colonization phenomena were detected over a period stretching from March 2023 to April 2024, demonstrating the efficacy of the biological risk control strategies applied. The results contained in this study was fundamental for the setup of the site management plan, which contains prescriptions regarding environmental and illuminance parameters, numbers of visitors and visit duration and pathways, the identification of critical areas to be monitored by restorers and frequency of inspections, and treatment procedures diversified according to different biological patina morphologies and compositions.

## 5. Conclusions

In conclusion, the study performed in the Roman Archeological Museum of Positano deepens the knowledge of the biological ecosystem of the hypogeous Roman *villa*. In particular, not only patinas with common morphology composed of fungi or green algae but also peculiar alterations mainly composed by bacteria were observed on the surface of Roman mural paintings. Interestingly, for some of the identified bacteria (*Ca*. Halysiosphaera, *Hyphomicrobium*, and *Amaricoccus*), this work represents the first report of their presence on wall paintings.

The three-year work allowed focusing not only on the characterization of biological alterations but also on drawing up efficient strategies and protocols for biodeterioration control and monitoring the efficacy of direct and indirect interventions. This led to the development of a specific maintenance program that must be applied over time in order to preserve the painted surfaces of the site and guarantee their fruition to the general public.

## Figures and Tables

**Table 1:** Description of the macroscopic aspects of the analyzed biological patinas.

Acronym	Description
FW	Filamentous white patina of considerable thickness (Figure 1a,b)
BS	Black spots adhered and partially interpenetrated into the inorganic substrate (Figure 1c,d)
BF	Black filamentous patina present on the painted surface (Figure 1e,f)
GP	Green patina scarcely adhered to the surface and of different thickness (Figure 2a)
FT	Soft, thin filamentous white patina (Figure 2b,c)
BsF	Brown semitransparent film of a sticky substance with a corrugated superficial morphology well adhered to the surface (Figure 2d)
GC	Granular clumps of incoherent material loosely adhered to the surface, beige or reddish-brown in color (Figure 2e)
JC	Jelly clumps made up of a gelatinous, sticky, transparent substance, with colors ranging from light yellow to brown (Figure 2f)
CC	Compact clumps made up of well-adhered lenticular accumulations on the surface, with varying colors unrelated to the underlying surface color. The observed colors included yellow ochre, red, green, blue and gray-black (Figure 2g)

**Table 2:** Summary of the analytical methods applied during the different campaigns for the study of biological patinas. The method was selected depending on the type of alteration.

	Alterations								
	FW	BS	BF	GP	FT	BsF	JC	GC	CC
MO	x	x	x	x	x		x	x	x
MC	x				x				
NGS					x	x	x	x	x

MO: microscopic observation; MC: microbiological culture; NGS: next-generation sequencing.

**Table 3:** Results of ATP bioluminescence assays.

	Patina				
	GP	GC	JC	CC	BsF
UN (RLU)	7429	633	134	4861	291
TR (RLU)	83	4	5	14	236
% decrease	99.88	99.37	96.27	99.71	18.90

UN: untreated patina; TR: treated patina; RLU: Relative Light Units.

**Table 4:** Direct and indirect methods developed to face biological colonization.

Direct Methods	
Fungal patinas disinfection	Biocide (Preventol RI50 5% in deionized water) applied twice by spraying.
Disinfection of CC, GC, JC, GP, BsF	Mechanical removal of part of the biomass and subsequent disinfection through a single application of the biocide (Preventol RI50 5% in deionized water) with a brush.
**Indirect methods**	
Temperature reduction and avoidance of temperature excursions	From 18 °C to 15 °C
Decrease of illuminance values on painted surfaces	From 120 lux to 50 lux
Reduction of the light income (openings and windows)	Application of specific filters on each opening of the site
Reduction of the thermohygrometric instability of the hypogeum	Closing of the entrance door to the crypt

## Data Availability

The raw reads are available at the NCBI Sequence Read Archive (SRA) database (SRA accession no.: PRJNA1129806).

## Supplementary Materials

The following supporting information can be downloaded at: https://www.mdpi.com/article/10.3390/microorganisms12081520/s1, Figure S1: Rarefaction curve based on Chao1 (a) and Shannon indexes (b); Figure S2: Evaluation of microbial beta-diversity. (a) displays the PCoA (Principal Coordinate analysis) of the bacterial community composition in samples; (b) displays the PCoA of the fungal community composition in samples.
