# Supplementary material for: The Hypogeous Roman Archeological Museum of Positano: Study of the Evolution of Biological Threaten and Development of Adequate Control Protocols
Source: Microorganisms. 2024 Jul 24;12(8):1520. doi: 10.3390/microorganisms12081520 (PMC11356633; doi:10.3390/microorganisms12081520)
Supplement: Supplementary file 1 [file microorganisms-12-01520-s001.zip › microorganisms-3106986-supplementary.pdf]

The hypogeous Roman Archeological Museum of Positano: study of the evolution of biological threaten and development of adequate control protocols

Federica Antonelli [1,2,*], Sara Iafrate [3], Marco Tescari [4], Manuel Giandomenico [3,5], Alma Kumbaric [1,6] and Marco Bartolini [1]

1 Biology laboratory, Istituto Centrale per il Restauro, via di San Michele 25, 00153 Rome, Italy; fedantonelli@gmail.com; alma.kumbaric@cultura.gov.it; marco.bartolini@cultura.gov.it
2 Bio.Co.Ré. lab, via Reatina, 10, Scurcola Marsicana, 67068, L'Aquila, Italy; fedantonelli@gmail.com
3 Mural paintings laboratory, Istituto Centrale per il Restauro, via di San Michele 25, 00153 Rome, Italy; sara.iafrate@cultura.gov.it
4 Biology laboratory, Istituto Centrale per il Restauro, Support staff Ales S.p.A., via di San Michele 25, 00153 Rome, Italy, marco.tescari@cultura.gov.it;
5 Department of Science of Antiquities, Sapienza University of Rome, Piazzale Aldo Moro 5, 00185 Rome, Italy; manuel.giadomenico@uniroma1.it
6 Department of Environmental biology, Sapienza University of Rome, Piazzale Aldo Moro 5, 00185 Rome, Italy

* Correspondence: fedantonelli@gmail.com

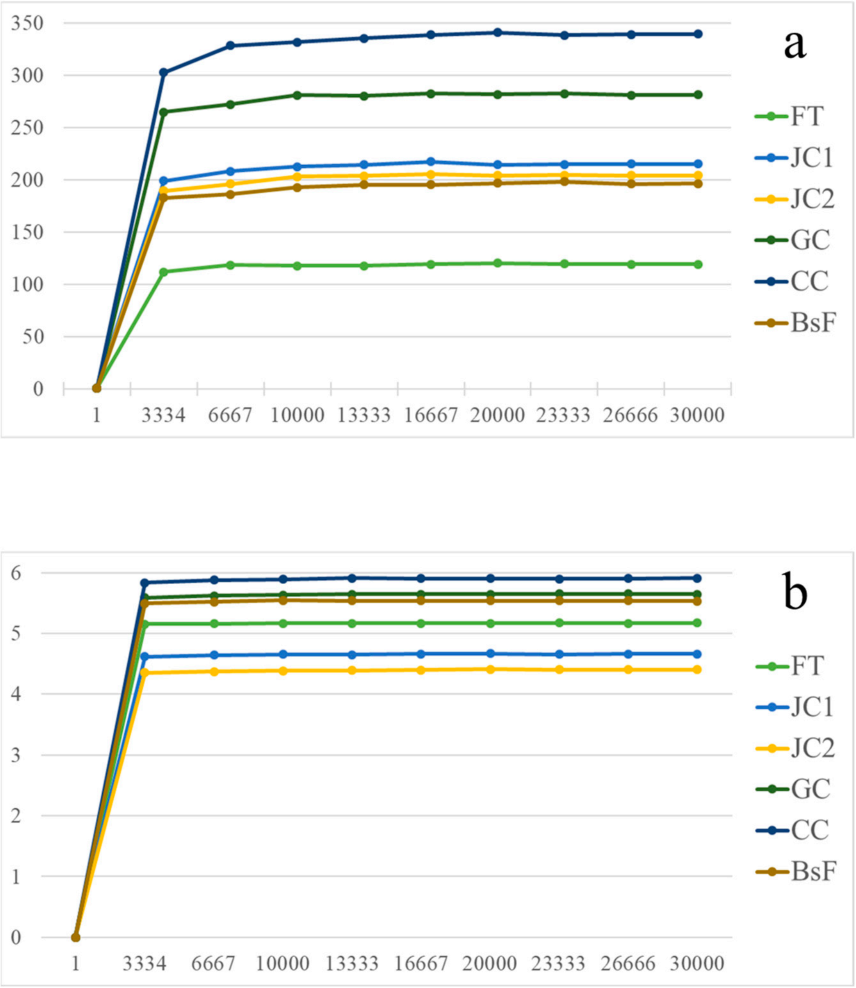

Figure S1. Rarefaction curve based on Chao1 (a) and Shannon index (b)

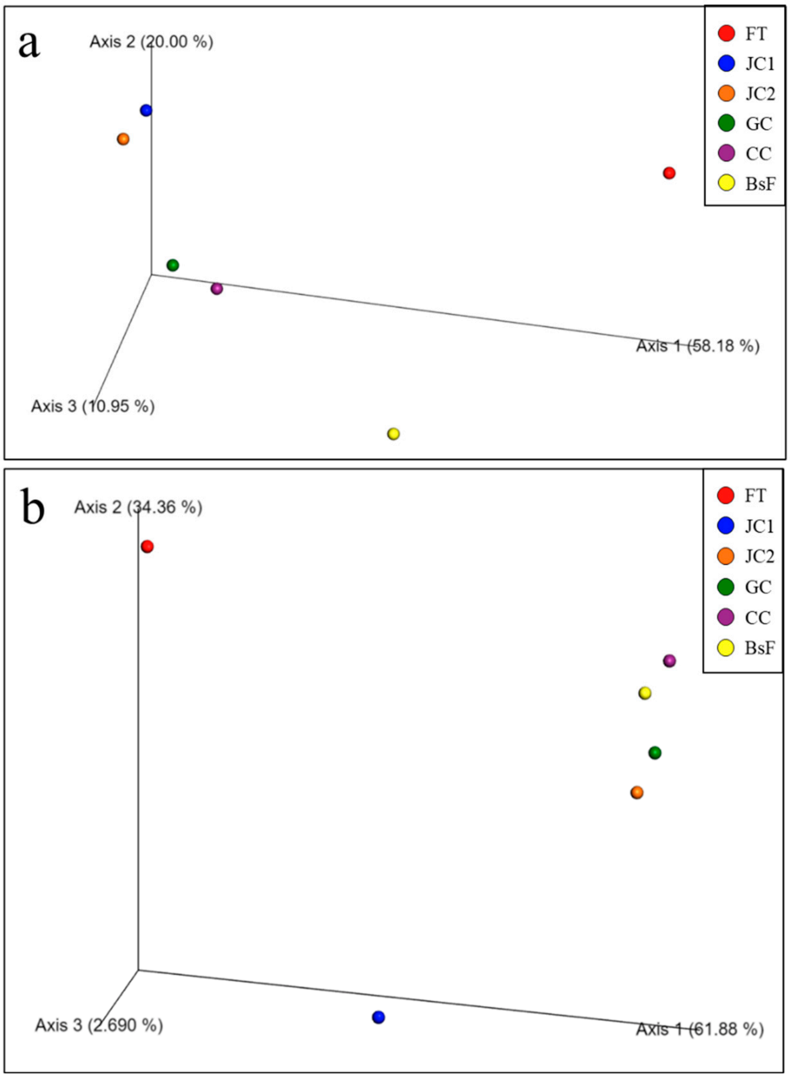

Figure S2. Evaluation of microbial beta-diversity. (a) displays the PCoA (Principal Coordinate analysis) of the bacterial community composition in samples; (b) displays the PCoA of the fungal community composition in samples.
